# Supplementary material for: Developing the Mental Health Sensitive School Concept: Insights from focus group discussions
Source: PLOS Ment Health. 2025 Jul 16;2(7):e0000368. doi: 10.1371/journal.pmen.0000368 (PMC12798506; doi:10.1371/journal.pmen.0000368)
Supplement: Attachment — Submitted filename: Rebuttal Letter.docx [file pmen.0000368.s002.docx]

Dear esteemed Reviewers,

We are highly grateful for your feedback, questions, and proposals. The following table lists the changes we implemented in the text and our responses.

Reviewer 1:

Thank you for the comment. However, the researcher's background is presented in the relevant section of the article “Researchers’ characteristics and reflexivity” to the extent that is necessary for the article format. A more detailed presentation did not seem “humble” enough.

| Concerns | Authors Replies and Changes made in the text |
| --- | --- |
| The introduction presents a rationale for the study, outlines the key research goals, aims and objectives. The description of the MHSS framework remains somewhat vague to the reader and (1) a more thorough description on what the model concretely entails would benefit the manuscript. | The detailed description of the MHSS, in its final form, is presented in the Discussion. We have had a lot of discussions on how to deal with these issues, as presenting the final version in the Introduction might be too early, but not presenting it at all will create confusion for the reader. So, thanks to your idea, we added the sentence in the Introduction: “The detailed description of the MHSS in its current form is presented in the Discussion section and Figure 1.” |
| Relatedly, (2) key terms should be defined: terms such as psychological support, mental health support, mental health and psychosocial support, mental health, psychosocial support are used throughout the manuscript and also by participants. It is unclear for the reader what different levels of support the terms represent or if the terms are used interchangeably. Do some of the terms refer for example to clinical psychological care for mental disorders, and other to psychosocial support to manage everyday stress? | Thank you for noticing the issue. We agree completely and have therefore implemented such changes:   - “psychosocial support” was deleted from the text, as it was just used occasionally as a synonym of “mental health support”. - “psychological support” was used in 2 contexts: 1) by us while referring to the official documents that use the term broadly, including in “psychological support” the mental health clinical and non-clinical support, as well as all tasks related, for example, to the support sutends in educations, resoving conflicts etc. 2) by FGDs participants, refereing to it in the most of the cases as support provided to deal with mental health problems (so, basically, mental health support). However, now we see in the text that this is not clear to readers, and we also followed the FGD participants and used the term inappropriately.   To avoid the confusion, we added the definition in the Introduction section (footnote) and changed the wording in other parts. |
| Throughout the manuscript, claims are made that (3) should be linked to earlier research (references). | Well noted, additional references are added and integrated into the Reference list |
| (4) The sampling techniques should be described in greater detail, for example how participants were recruited. | Well noted, information is added to the Sampling strategy section of the article. |
| I applaud the authors’ attention to power dynamics, safety and respect for the participants, however, in relation to the overall manuscript these descriptions seem lengthy. | In this case, we followed the SRQR guideline. Unfortunately, there is sometimes a problem with the conflicting demands on the guidelines side, and volume limitation on the journals side ☹ We tried to do our best to bridge them. |
| There are three authors to the paper, however, the methods section mentions that “multiple” researchers conducted the thematic analysis. | Weel noted, we changed the wording from “multiple” to “All three researchers…” |
| (5) I recommend describing somewhat more in detail the numbers and backgrounds of the researchers, although not necessarily to an individual level. | Thank you for the comment. However, the researcher's background is presented in the relevant section of the article “Researchers’ characteristics and reflexivity” to the extent that is necessary for the article format. A more detailed presentation did not seem “humble” enough. |
| The awareness of assumptions and bias is mentioned, but bias are not critically discussed and analyzed, neither in the limitation section (6). | Thank you, relevant sub-section is added and Limitation section is extended. |
| Comments regarding the discussion (7): The identified themes cover the field of MHPSS comprehensively and, perhaps given the constraints of space, the discussion of each of them remains superficial. It is unclear for the reader how the MHSS framework was developed based on the FGD input given that the original framework was not described in detail. The description of the finalized MHSS framework remains somewhat unattached to the input from the FGDs. | Well noted, linkage paragraph is added to the dicussion section. |
| Further, the input is not critically discussed in light of existing literature; for example, there are findings indicating that mental health awareness may be counterproductive in certain conditions. | Thank you, relevant paragraph is added to the dicussion section. |
| Minor: p. 11 The manuscript says that special attention was given to anonymity, however, the FGD is said to have started with introductions. | Thank you, clarification is added: (therefore, during the transcription, all personal information was not included). |

Reviewer 2:

It is absent, I think, due to the timing of the study; participants were so focused on the immediate reaction and support, that touching issues you mentioned were outside their (and our as researchers) capacity.

| Concerns | Authors Replies and Changes made in the text |
| --- | --- |
| I feel like perhaps there is not enough touching on the impacts of the ongoing conflict and how this influences every stakeholders' answers to the focus groups? | Thank you, we now added the text that is addressing this in the Dicsussion section. |
| Ln 160 - you have "to balance these perspectives" twice in the same sentence. | Thank you, corrected |
| Results Ln 409 - adaptation to crisis context. I understand that being ready to react adequately in the context of an attack is paramount. However, I wonder whether there is space/room for children and teachers to simply talk about how difficult it is to be facing war (and to have been for so long?). Do they have opportunities to explore existential issues? To talk about the impact the war has had on their mental health? | It is absent, I think, due to the timing of the study; participants were so focused on the immediate reaction and support, that touching issues you mentioned were outside their (and our as researchers) capacity. |
| Discussion -Is there space for the war (addressing its effect, talking about it, etc.) in the MHSS pyramid? It seems like the impacts of this conflict will continue to reverberate for many years, possibly decades (even once the conflit is over), perhaps it should be added in there? | Can`t agree more! Thank you for bringing it in. We added relevant text to the Discussion section. |
